# Psychological Violence in Image-Based Sexual Abuse (IBSA): The Role of Psychological Traits and Social Communications—A Narrative Review

**DOI:** 10.3390/healthcare13172083

**Published:** 2025-08-22

**Authors:** Carmela Mento, Martina Praticò, Maria Catena Silvestri, Clara Lombardo, Francesco Pira

**Affiliations:** 1 Department of Biomedical, Dental Sciences and Morpho-Functional Imaging, University of Messina, 98122 Messina, Italy; cmento@unime.it (C.M.); martinapratico@hotmail.com (M.P.); mariacate@libero.it (M.C.S.); 2 Department of Health Sciences, “Magna Graecia” University of Catanzaro, 88100 Cantanzaro, Italy; 3 Department of Ancient and Modern Civilizations, University of Messina, 98122 Messina, Italy; francesco.pira@unime.it

**Keywords:** non-consensual intimate images, technology usage, social withdrawal, non-consensual pornography, victim blaming

## Abstract

This narrative review has examined the psychological and emotional effects of non-consensual distribution of intimate images, a phenomenon known as Image-Based Sexual Abuse (IBSA). It is an increasingly frequent phenomenon, and in detail, it consists of when a person uploads nude/semi-nude photos of someone online; therefore, it is similar to nonconsensual pornography. According to the scientific literature, this phenomenon has caused victims to experience trust issues, anxiety, depression, suicidal thoughts, social withdrawal, public shame and humiliation, depression, fear, anxiety, stress, inability to find new romantic partners, and several other mental health effects. The aim of this study was to study the psychological consequences of Image-Based Sexual Abuse on victims. This study is a narrative review conducted in accordance with the SANRA guidelines. Findings have shown that revenge pornography and non-consensual intimate images have had devastating impacts on victims’ mental health.

## 1. Introduction

The Internet is an invaluable resource, but if used with little responsibility and awareness it is capable of causing serious damage to human mental health [1,2]. With the recent spread of social media and technology, sending, producing and distributing photographs and using webcams in communication have become common. While this transformation of digital interaction offers new forms of intimacy and self-representation, it has also introduced unprecedented risks, particularly when consent is ignored or violated.

### 1.1. Image-Based Sexual Abuse (IBSA) and Revenge Porn

Revenge pornography is a category of online pornography usually created with the consent of those depicted, but then distributed without their consent [3]. Image-Based Sexual Abuse (IBSA) is defined by Powell and colleagues [4] as the non-consensual creation, distribution, and/or threat to distribute intimate or sexual materials (i.e., images, videos, or texts). This phenomenon is a serious criminal offense, as it is non-consensual pornography and involves uploading nude or semi-nude images and/or videos of a person online without their consent [2,5]. An important condition is correlated with IBSA and occurs when a person uploads nude/semi-nude photos of someone online, often as revenge after a relationship has ended. Image-Based Sexual Abuse is becoming an increasingly common form of violence. Although there are numerous benefits that humans can obtain from technology, it must be admitted that not everyone is able to make a benevolent use of it [6]. Today the consequences of IBSA are affecting scientific research, given its exponential increase, and although many do not recognize this form of sexual abuse as such, the effects are very clear and harmful [7]. The international literature on Image-Based Sexual Abuse (IBSA) highlights a significant prevalence worldwide. A multinational study conducted on a large sample across ten countries reported that 22.6% of participants had experienced forms of IBSA, with higher rates among LGBTQ+ individuals and young adults; while men and women were victimized at similar levels, women reported more severe psychological and emotional consequences [8]. Cultural and legislative differences across countries appear to influence perception, reporting, and support: in the United Kingdom and Australia, specific laws criminalizing IBSA have been in place for several years, whereas in Italy such legislation is more recent. On a European level, only in 2024 did the European Union adopt the Directive on Violence against Women and Domestic Violence, marking the first binding EU legislation aimed at addressing various forms of sexual and gender-based violence, in conjunction with regulatory instruments such as the Digital Services Act and the AI Act [9].

### 1.2. Digital Abuse as Psychological Violence

Image-Based Sexual Abuse is performed as a revenge act on the part of an ex-partner, whose purpose is probably to restore (alleged) superiority within a couple dynamic or simply towards the woman. Often there is the belief that the only possible form of violence—or in any case, that it can cause concrete damage to the victim—is physical, but the scientific literature does not prove this: in 1987, Liz Kelly [10] developed the concept of a continuum of violence by referring to a set of abuse, threats, and harassment that easily intersect each other, making it impossible to draw a clear distinction. However, these malicious actions have in common the same purpose: to use bullying, force, and oppression to have full control of a woman [11].

### 1.3. Psychological Aspects

Powell and colleagues [12] recently studied the psychological consequences of adults who suffered IBSA and found that a high percentage of participants shared some nude images or of sexual nature with their partner, and a higher percentage of sample had suffered threats to disseminate their nude images or images of a sexual nature without the consent of the person depicted, with serious consequences for the victims such as depression, anxiety, social withdrawal, and suicide risk.

Since 2019, the Italian regulation system has identified this problem as a crime, introducing the so-called “Red Code”. Users who spread this material are former partners of the victims who wish to humiliate them after the non-consensual breakdown of the relationship between the two [2]. For this reason, according to Mcglynn and colleagues [13] it would be more appropriate to talk about sexual violence based on image or sexual abuse facilitated by technology. The literature highlights that Image-Based Sexual Abuse perpetrators (IBSA) possess specific psychological traits, including dimensions of the Dark Triad such as Machiavellianism, Narcissism, and Psychopathy, as well as tendencies towards moral disengagement and sexist attitudes [14]. In this study, the Machiavellianism and Psychopathy variants predicted a higher propensity of IBSA, while the Narcissistic variant correlated with feelings of arousal and enjoyment related to abuse. In parallel, victims of IBSA report high levels of anxiety, depression, posttraumatic symptoms, and shame, often associated with a perception of devaluation and self-condemnation [15]. Furthermore, the literature emphasizes the crucial role of communicative dynamics such as coercive sexting, non-consensual sharing on social media, and the emergence of public discourses that stigmatize victims [16]. These communicative practices increase the psychological impact through the processes of social blaming and emotional manipulation.

### 1.4. The Role of Sexting in IBSA

The study by Barrensense-Dias and colleagues carried out in 2017 recognized the activity of sexting as a forerunner of IBSA. Sexting is sending and receiving sexually explicit messages and/or multimedia content [17]. Two forms of sexting have been found: active and passive. The first refers to the act of creating, sending, or forwarding such content to third parties, and the second involves actions such as asking or receiving from the previous. The consequences of this consenting phenomenon could be revenge by the person who has been left. In fact, in IBSA the ex-partner uploads nude photos that were exchanged through sexting [5]. A recent and interesting systematic review of the phenomenon has been carried out by Paradiso et al. [18], who studied the phenomenon mainly in the following countries: United States, Canada, Australia, United Kingdom, and New Zealand. Furthermore, studies on sexting have reported correlations with risk behaviors linked to alcohol or substance use (e.g., impulsivity and reduced inhibition). In particular, Dir et al. [19] found that sexting mediates the relationship between alcohol use and the risk of sexual assault, and that sex- and alcohol-related expectancies mediate the association between problematic alcohol use and sexting. Although direct research on IBSA in this area remains limited, these findings suggest that substance use, or new psychoactive substances (NPS), may facilitate non-consensual online sexual behaviors.

Pina and collaborators [7] showed that revenge porn is a phenomenon with several implications, and it has devastating effects on individual vulnerability. In fact, several studies have testified that the psychological consequences of victims of revenge are comparable to those of traditional sexual violence. Finally, the dark web represents a particularly concerning context: some anonymous and poorly regulated platforms facilitate the sharing and dissemination of non-consensual intimate images, making the monitoring of such content and the protection of victims extremely challenging [20]. This review has focused on a reality that places the victims as protagonists of double violence: violated in their own intimacy and at the mercy of the judgment of others, a double-stranded situation that alters mental health, with the involvement of traumatic existential aspects of human life. This narrative review aims to analyze the psychological impact of IBSA on victims, the psychological characteristics associated with perpetrators and victims, and finally, the role of social and communicative dynamics—including sexting, digital interactions and societal responses—in the perpetuation of this form of violence.

## 2. Methods

This study is a narrative review, conducted following the SANRA (Scale for the Assessment of Narrative Review Articles) guidelines.

### 2.1. Research Question and Objectives

The research question for the narrative review was: “what are the psychological consequences of Image-Based Sexual Abuse (IBSA), and what psychological traits and social communication factors are involved in victims and perpetrators?”

### 2.2. Sources of Information

The literature search was conducted across two databases (PubMed and Scopus) and complemented by gray literature (e.g., institutional reports and policy documents) when relevant.

### 2.3. Time Frame and Language

Articles written in English or Italian from 2020 to 2025 were analyzed,.

### 2.4. Search Strategy

The search was performed using keywords combined with Boolean operators “AND”, including: “Revenge porn”, “Image-Based Sexual Abuse”, “Non-consensual pornography”, “Sexting”, and “Online sexual victimization”.

### 2.5. Eligibility Criteria

#### 2.5.1. Inclusion Criteria

Articles were included in the review according to the following inclusion criteria: articles written in the English or Italian language, and containing quantitative and qualitative information on revenge porn, Image-Based Sexual Abuse, and non-consensual pornography. References from the selected articles were also checked.

#### 2.5.2. Exclusion Criteria

Articles were excluded if irrelevant to the examined topic. Reviews, case reports, case-series, and articles on animal models were also excluded.

### 2.6. Selection Process, Data Extraction and Synthesis

Articles were analyzed considering keywords, methodology, constructs, and finally, variables to then summarize the most important data, including the purposes of the piece of research, the sample selected, method, and results obtained. The synthesis was integrative and thematic, organized by conceptual areas. All the articles were then compared, interpreting the results to be able to recognize the main issues and divide them by similarity. The selection was conducted by a single reviewer, and any uncertainties were discussed within the research group.

### 2.7. Risk of Bias

A formal risk of bias assessment was not conducted, as it was not required by the narrative design of the review. The included studies were selected and synthesized based on their relevance to the research question and their contribution to the thematic analysis, without the application of standardized evaluation tools.

## 3. Results

Seventeen articles examined psychological violence in Image-Based Sexual Abuse (IBSA) (Table 1). The analysis of the literature confirms that the phenomenon of revenge porn and the non-consensual distribution of intimate images constitutes a form of Image-Based Sexual Violence, with consequences for the psychophysical well-being of victims. Several studies have highlighted the emotional and psychological difficulties linked to the experience of revenge porn and non-consensual sexting. The study of Bates [5], through a qualitative analysis conducted on 16 women victims of illicit sharing of intimate material, identified significant psychological distress, including anxiety, depression, chronic stress, dysfunctional coping strategies, and suicidal ideation. Similarly, Chaudhary et al. [21] revealed that adolescents involved in sexting reported experiencing sadness, anxiety, anger, stress, and lowered self-esteem. Gassò et al. [22] found that one-third of the youth sample had engaged in active sexting, with 3.3% reporting victimization through illicit dissemination of intimate images. Male victims showed higher levels of depression, indicating a gendered dimension in the psychological impact of digital abuse, while general psychopathology and anxiety levels did not differ significantly by gender. A few studies have focused on identifying psychological factors and personality traits that increase vulnerability to both victimization and perpetration in the context of Image-Based Abuse.

Brenick et al. [23] showed that anxious attachment and rejection sensitivity facilitate victimization, suggesting an emotional dependency that can be exploited through coercive practices. Avoidant attachment, in contrast, was linked to a more detached perception of sexting. Brewer et al. [24] and Pina et al. [7] found that “Dark Triad” traits (Narcissism, Machiavellianism and Psychopathy) negatively affect romantic relationships and are linked to revenge porn behavior. Brinkley et al. [25] explored the link between sexting and borderline personality disorder (BPD), suggesting that this behavior contributes to adolescents’ psychological distress. Research suggests that sexting at the age of sixteen may be linked to risky sexual behaviors, and in adulthood to traits of borderline personality disorder. Gamez-Guadix et al. [26] identified positive correlations between sexting and traits such as neuroticism and extroversion, and negative correlations with agreeableness and conscientiousness. Wissing and Reinhard [27] showed that while no significant correlation was found between the Dark Triad and deception detection abilities, Psychopathy was associated with judgments of trust. Other studies have examined the social and cultural dynamics related to the practice of sexting and the public perception of revenge porn. Casas et al. [28] showed that among adolescents, sexting is influenced by social variables such as the need for popularity and cyber-gossip, with significant gender differences: girls receive more negative evaluations than boys. Gewirtz-Meydan et al. [29] reported that although 86% of participants considered sexting illegal under the age of 18, those who engage in it tended to minimize the risks. Girls were more aware of the possible negative consequences. Klettke et al. [30] declares that it would be more appropriate to adopt an integrative perspective in which a continuum characterized by normative sexting behaviors is revealed, and therefore aimed at a plausible sexual curiosity, up to the definitive coercive one which is correlated to negative repercussions on mental health. Several pieces of evidence testify a link between sexting and online victimization. However, the phenomenon, defined as a form of sexual interaction, is not necessarily set as risky behavior but it has been shown that it could lead to phenomena of sextortion, cyberbullying or even illicit dissemination of intimate material. Scott and Gavin [31] and McKinlay and Lavis [32] emphasized that sexting is perceived as particularly risky by women, and that the clothing of the victims in the images shared influences judgements of guilt. In particular, 80% of the sample admitted to sending or receiving intimate images. Powell et al. [4] found that the majority of perpetrators were male and the majority of victims female, often ex-partners or people with whom there had been an intimate connection, indicating that Image-Based Sexual Abuse can be classified as a form of domestic or intimate partner violence. In line with this, a related study conducted in Spain looked at 210 men who had committed intimate partner violence and were sent to a treatment program. The researchers used a known classification to divide them into three groups: family-only, borderline/dysphoric, and generally violent/antisocial. They found that men in the generally violent/antisocial group were more likely to drop out of the program and commit violence again, followed by those in the borderline/dysphoric group, and finally the family-only group. This suggests that understanding different personality types can help make intervention programs more effective, especially when Image-Based Abuse happens in intimate relationships [11]. Finally, Wood et al. [33], in a comparative study across five European countries, showed that sexting increases with adolescent age, and is associated with the Dark Triad and a tendency to share intimate content without consent. Among the studies reviewed, some key characteristics of psychological violence emerged: coercive control through the threat of exposure, emotional manipulation within intimate or former relationships, public humiliation facilitated by digital platforms, and loss of autonomy and privacy. Despite the methodological diversity of the studies, the psychological and relational nature of the harm inflicted by Image-Based Sexual Abuse (IBSA) is emphasized.

Overall, the literature provides a complex and multifaceted view of the phenomenon, emphasizing the interplay between psychological, relational, and cultural factors that contribute to its prevalence and impact.

**Table 1:** Psychological violence in Image-Based Sexual Abuse (IBSA).

Authors	Title	Aim	Sample	Materials and Measures	Results
Bates, (2017) [5]	Revenge Porn and Mental Health: A Qualitative Analysis of the Mental Health Effects of Revenge Porn on Female Survivors	This research investigates the emotional and mental health consequences experienced by female survivors of revenge porn.	18 female revenge porn survivors	Semi-structured interviews	Women have experienced problems with confidence, anxiety, depression, the use of dysfunctional coping mechanisms, a decline in self-esteem and loss of control.
Brenick et al., (2017) [23]	Victimization or Entertainment? How Attachment and Rejection Sensitivity Relate to Sexting Experiences, Evaluations, and Victimization	This chapter provides an overview of current research on how emerging adults engage in and evaluate sexting, with particular attention to the potential risks of becoming victims of sexting.	169 EAs (the social lives of emerging adults)	Revised Adult Attachment Scale, Adult Rejection Sensitivity Questionnaire, the sexting questionnaire of Alderson and Samimi	Anxious attachment together with avoidant attachment and susceptibility to rejection are facilitating factors of sexting and victimization. Those who presented with avoidant attachment considered sexting to be a little fun experience as opposed to those with anxious attachment.
Brewer, et al., (2018) [24]	Dark Triad and romantic relationship attachment, accommodation, and control	These studies explored the impact of Dark Triad personality traits (Machiavellianism, Psychopathy, and Narcissism) on the dynamics of women’s romantic relationships	Study 1: women (N = 122). Study 2: women (N = 265). Study 3: women (N = 240)	Mach IV, Levenson Self-Report Psychopathy Scale, NPI-16, Experiences in Close Relationships Revised Questionnaire, Dark Triad trait measures, the Accommodation Scale and Interpersonal Violence Control Scale.	The results show that the traits of the Dark Triad (Narcissism, Machiavellianism and Psychopathy) affect attachment, accommodation and control.
Brinkley et al., (2017) [25]	Sending and receiving text messages with sexual content: Relations with early sexual activity and borderline personality features in late adolescence.	The study examined adolescents’ sexually explicit written messages to investigate the associations between sexting, sexual activity, and features of borderline personality disorder.	Participants included 181 10th grade adolescents (85 girls and 96 boys, 15–16 years old)	The Romantic Relationships Questionnaire (RRQ), Dating Questionnaire, The Romantic History Questionnaire, McLean Screening Measure for BPD (MSI-BPD)	The results confirmed the hypothesis that sexting practiced at the age of sixteen is related to sexual activity and risky sexual behavior along with borderline personality traits at eighteen.
Carbajosa, et al., (2017) [11]	Differences in treatment adherence, program completion, and recidivism among better subtypes	This study aimed to cross-validate Holtzworth-Munroe and Stuart’s typology using a Spanish sample of court-referred perpetrators of intimate partner violence	The sample consisted of 210 batterers court referred to a batterer intervention program.	Millon Clinical Multiaxial Inventory- III, The Revised Conflict Tactics Scale (CTS- 2), Spousal Assault Risk Assessment Guide, Plutchik’s Impulsivity Scale, Alcohol Use Disorders Identification Test (AUDIT), State-Trait Anger Expression Inventory (STAXI-2)	The study confirmed three abuser subtypes predicting treatment outcomes and recidivism. Generally violent/antisocial men showed the worst results, while family-only had the best. Findings support tailored interventions based on abuser profiles
Casas et al., (2019) [28]	Exploring which factors contribute to teens’ participation in sexting	This study examines whether the need for popularity, involvement in cyber-gossip, social competence, normalization of sexting, and willingness to sext predict adolescents’ level of sexting participation, while also exploring the role of gender in this behavior.	1431 (46.4% female) Spanish adolescents, aged 11–18 years, participated in a two-wave longitudinal study (https://www.sciencedirect.com/topics/social-sciences/longitudinal-analysis, accessed on 16 April 2025) with a time lag of four months.	Normalization Sexting Questionnaire, The Need for Popularity Scale, the Cyber-gossip Questionnaire for Adolescents (GCQ-A) and Perceived Social Competence Scale (PSCSII).	The study found that all examined factors predicted adolescents’ involvement in various sexting behaviors, but their influence varied by behavior and gender. For girls, cyber-gossip and need for popularity were most influential, while for boys, normalization of sexting and willingness to sext played a greater role. These findings underscore the need for gender-sensitive prevention strategies.
Chaudhary et al., (2017) [21]	Sexting and Mental Health: A School-based Longitudinal Study Among Youth in Texas	The current study, therefore, attempts to examine the empirical relationship between sexting and the mental health of youth, and to propose recommendations for interventions.	1760 sixth grade students from the 10 participating middle schools.	Generalized Anxiety Disorder-7 (GAD-7), Modified Depression Scale (MDS)	The study reveals how the practice of sexting is widespread among adolescents and is associated with problems of anxiety and depression.
Gámez- Guadix et al., (2017) [26]	Sexting among Spanish adolescents: Prevalence and personality profiles.		3.223 Spanish adolescents from 12 to 17 years of age (49.9% female)	The Sexting Questionnaire, The German Socio-Economic Panel (GSOEP) Big Five Inventory (BFI-S).	The findings show a growing prevalence of sexting among adolescents, likely due to increased social media use and a heightened focus on relationships during this developmental stage. The study also confirms significant associations between sexting and the personality traits of conscientiousness, extroversion, and neuroticism. Additionally, sexting was found to be more common among LGBT youth, highlighting a relevant demographic trend.
Gassò, et al., (2020) [22]	Sexting, Online Sexual Victimization, and Psychopathology Correlates by Sex: Depression, Anxiety, and Global Psychopathology	This study aimed to examine sexting behaviors, online sexual victimization, and their associations with mental health—specifically global psychopathology, anxiety, and depression—using clinically validated measures, with analyses conducted separately for men and women.	The sample comprised 1370 Spanish college students including 999 women (73.6%) and 359 men (26.2%)	Socio-Demographic Questionnaire, SCL-90, LSB-50 questionnaire, Juvenile Online Victimization Questionnaire (JOV-Q)	The results show that one third of the sample had been involved in active sexting, two thirds instead in passive, underlining a gender difference in this regard: men received more sexually explicit messages from women. A total of 3.3% of the sample was the victim of illicit dissemination of their intimate images. Men suffered more from depression than women but there are not significant differences between genders about anxiety and general psychopathology.
Gewirtz et al., (2018) [29]	What do kids think about sexting?	This study examined U.S. underage youths’ attitudes toward sexting and reporting it, and explored how various factors—such as age, sexual activity, identity, substance use, and media habits—relate to these attitudes.	1560 youth Internet-users, ages 10–17 years old, and a caregiver	Six questions were asked about the possible consequences of sexting, four questions about how they would respond to sexually explicit content, and an open question to investigate what the boy or girl meant by “naked” or “semi-naked”.	86% of the sample considers sexting an illegal activity if practiced under the age of 18. On the other hand, young people who had practiced it did not believe that it was a crime or that it could damage their reputation. Girls are more aware of the possible negative consequences than boys.
Klettke et al., (2019) [30]	Sexting and Psychological Distress: The Role of Unwanted and Coerced Sexts	Assess whether sexting may be associated with poorer mental health.	444 young adults	Semi-structured interviews	Sexting was associated with high levels of psychological distress, low self-esteem and stress.
Mckinlay & Lavis (2020) [32]	Why did she send it in the first place? Victims blame- context of ‘revenge porn’	This research investigated the perceptions that individuals form about ‘revenge porn’ victims, aiming to gain more understanding from a victimization perspective as a first step towards improving victim outcomes.	N = 122 (female 76%)	The Sexual Double Standard Scale (SDSS)	The research shows that the more the victims were naked in the images released without their consent, the more they are considered guilty. A total of 80% of the sample admits having received or sent an intimate image demonstrating how the phenomenon is constantly expanding
Pina et al., (2017) [7]	The Malevolent Side of Revenge Porn Proclivity: Dark Personality Traits and Sexist Ideology	Exploring a form of technology facilitated sexual violence (TFSV) known as revenge porn.	One hundred adults, aged 18–54	Ambivalent Sexism Inventory- Short Version (ASI), Short Dark Triad (SD3), Comprehensive Assessment of Sadistic Tendencies (CAST), The Revenge Porn Proclivity Scale.	Most of the sample reveals that they do not commit an act of Revenge Porn but highlight approval of the behavior itself. The traits of Machiavellianism, Narcissism and Psychoticism are related to pornographic revenge behaviors.
Powell et al., (2019) [4]	Image-based sexual abuse: The extent, nature, and predictors of perpetration in a community sample of Australian residents	The study aimed to examine: the extent of self-reported IBSA perpetration, the nature of self-reported IBSA perpetration, and the predictors of self-reported IBSA perpetration.	4053 Australian residents, 2298 females and 1755 males, with an average age of 34.55 years.	Demographic characteristics; Sexual image-based abuse myth acceptance; Online dating behaviors; Sexual self-image behaviors; IBSA victimization; IBSA perpetration and the nature of IBSA perpetration.	The results show that the perpetrators were mostly male, and the victims were female who were former partners or people with whom an intimate bond was established. Sexual abuse based on image is configured as harassment in the family and intimate context.
Scott & Gavin (2018) [31]	Revenge pornography: the influence of perpetrator-victim sex, and observer sexting experience on perceptions of responsibility.	This paper aims to explore how the sex of the perpetrator and victim, the sex of the observer, and the observer’s experience with sexting influence perceptions of seriousness and responsibility in cases of revenge pornography.	239 students (120 men, 119 women) with an average age of 20.13 years	The sample filled out a questionnaire with a hypothetical scenario (which represents the different levels of perpetrator-victim sex), five items on their perceptions of the situation subjected to (seriousness and responsibility), five items on personal experiences of sexting and three questions on demographic information (gender, age and university course)	40% of the participants practiced sexting stating that it was part of the courtship. Furthermore, women perceived possible risks more than men. Finally, those who had practiced sexting tended not to blame the victim if there was illicit dissemination of intimate material.
Wissing & Reinhard (2017) [27]	The Dark Triad and the PID-5 Maladaptive Personality Traits: Accuracy, Confidence and Response Bias in Judgments of Veracity	In this study, were investigated the relationships between the Dark Triad traits, maladaptive PID-5 personality traits and the lie-detection process	207 participants (59.9% female)	Naughty Nine short scale, Dark Triad traits, the German version of the Personality Inventory for DSM-5 Brief Form (PID-5-BF).	The results show an absence of correlation between the Dark Triad and the ability to detect deception, while there was one between Psychopathy and the judgments of trust.
Wood et al., (2015) [33]	Images across Europe: sending and receiving sexual images and associations with interpersonal violence in young people’s relationships		4564 young people aged between 14 and 17	Survey that contains questions about demographic characteristics, physical, emotional online and offline, sexual violence, the sending and receiving of sexually explicit messages or content, their impact and the sharing of sexual images.	The phenomenon of sexting grows with advancing adolescence. Research took place in five different European countries: Cyprus, England, Norway and Italy. Sexting is not practiced in Cyprus, probably due to the recent advent of technology. The impact of sexting is both positive and negative.

## 4. Discussion

This review has examined the recent international scientific literature related to the phenomenon of revenge pornography and non-consensual distribution of intimate images, and the consequences on victims’ quality of life and health. The articles examined have been published since 2015, although the topic has become popular in previous years. Cases of revenge pornography and non-consensual distribution of intimate images mainly affect women and almost always have sexting as a precursor. Sexting is defined as sending multimedia content with a sexual background [12]. For this reason, this review has focused on its description: it has therefore been a source of interest for several empirical studies. Adolescents are more involved in this practice, and the study highlighted that for women it is a behavior strongly correlated to social image, while for men it reveals itself as an important modality of approach from a sexual point of view [28]. The consequences of revenge pornography and non-consensual distribution of intimate images are different in people: in the last case there is mental pain, negative emotional consequences and damage to self-image; therefore, an underlying sexist basis of this phenomenon can be deduced [33,34].

Given the wide spread of the phenomenon, it was equally necessary to focus on the consequences of this phenomenon on mental health, and the results show how this is connected to states of anxiety and depression [21].

The link between personality traits and sexually explicit messages has also been a cause for investigation; research carried out on adolescent subjects illustrates that conscientiousness, neuroticism and extroversion are connected to the implementation of sexting [26]. As widely claimed, sexting is not a crime, but a totally regular practice that can characterize a more or less stable love relationship. Notwithstanding this assumption, it is worth noting that sexting under the age of 18 is, in some contexts, classified as illegal, although this may not be readily perceived by the individuals directly involved in such practices [29]. Cases of illegitimate spread of previously created and owned content within the intimacy of a couple can derive from this type of behavior; in fact, a study carried out in 2020 showed that 3.3% of the sample had been a victim of this offense [22]. Revenge porn is sharing intimate material without the consent of the person depicted. The spread has the purpose of damaging the reputation of the person; this problem is in turn part of the broader framework of non-consensual pornography (NCII), in which online publication of sexually explicit contents takes place for various reasons, such as fun. Global data confirm the pervasiveness of NCII. A recent multinational study involving more than 16,000 adults in ten countries reported that 22.6% had experienced at least one form of Image-Based Sexual Abuse, with higher rates of victimization among LGBTQ+ individuals and young adults; women reported more severe psychological impacts than men, and almost a third did not report their experience. In England and Wales, approximately 7 per cent of women and 11 percent of men aged 18–34 reported having been threatened with the publication of intimate images [35].

Several studies have investigated the personological characteristics capable of facilitating the implementation of such harmful actions; the first study that gave the input for further investigations dates back only to 2017, and took place in the English territory, demonstrating that the perpetrators of cyber-rape not only have a strong correlation with the Dark Triad (Machiavellianism, Psychopathy and Narcissism), but also approve of the behavior itself [7].

Although revenge porn is widely minimized, its effects on mental health and quality of life leave indelible traces in those who are victims: empirical studies have testified that the consequences are comparable to those of typically known physical violence. The pattern of anxiety, depression, loss of control, low self-esteem, post-traumatic stress disorder, suicidal thoughts, extremely dysfunctional coping strategies and alcohol abuse, radically disrupt the everyday life of the women who suddenly find themselves imprisoned in a tunnel, in which the way out seems impossible to reach [5].

The concept of violence embraces several dimensions that are underestimated by a large part of the population. We are not faced with a unitary concept, which is why it is essential to discuss it in terms of a continuum that groups all those physical and/or psychological actions that have the attempt to damage the autonomy of women as their common denominator. Revenge pornography and non-consensual distribution of intimate images is recognized in all respects as a form of Image-Based Sexual Abuse that shows itself in a familiar scenario, so that most victims, as documented in a piece of research conducted in Australia, are women who receive this violence from one of their ex-boyfriends [4]. A consequence connected to pornographic revenge or—more generally—to sexual abuse is blaming victims: deeming the victim to be directly guilty of the harassment received. Current prevention and intervention strategies include the classification of offenders using the Holtzworth–Munroe typology, which has proven effective in Spain. However, personalized interventions and educational programs based on digital literacy have shown preventive potential [36].

## 5. Conclusions

This review shows that Image-Based Sexual Abuse (IBSA), including revenge pornography and non-consensual distribution of intimate images, is associated with significant psychological consequences, often comparable to those experienced by victims of sexual violence. The findings highlight the prevalence of emotional distress, anxiety symptoms, depression and post-traumatic stress among victims, underlining the impact on mental health. This study contributes to the understanding of IBSA, especially with regard to cross-cultural perspectives and longitudinal analyses. The literature review emphasizes the need for prevention strategies, support systems for victims, and developing educational interventions to promote digital ethics and respect for individual autonomy. Future research should expand interdisciplinary approaches and explore the legal, technological and media dimensions of IBSA to inform more effective responses and protective measures.

## 6. Limitations

This review presents several limitations. The main limitation lies in the narrative review approach to the available literature, which offers lower reproducibility compared to a systematic review. Nonetheless, its strength resides in contributing to the understanding of the complex and multifactorial dynamics underlying psychological violence in Image-Based Sexual Abuse (IBSA), with particular attention to the role of psychological traits and social communications. In addition, although SANRA guidelines were followed, a formal risk of bias assessment was not conducted, and expert judgment was not applied in evaluating the included studies. This choice, consistent with the narrative review design, could introduce a certain degree of subjectivity and a lower level of methodological rigor than systematic reviews.

Most of the studies come from English-speaking countries, particularly the United Kingdom, the United States and Australia, with limited representation from Southern or Eastern Europe. This limits the applicability of the findings to different cultural or legislative contexts. Most of the included studies are cross-sectional, which limits the ability to establish causal relationships between Image-Based Sexual Abuse and its psychological consequences. Furthermore, this study focuses mainly on the psychological dimensions, paying less attention to the legal, technological and media aspects that are equally crucial to comprehensively understanding and addressing the issue.

## Data Availability

All data supporting this narrative review are available in the original articles, reports, and preprints referenced throughout the manuscript.
